# Potential Protective Role of Amphibian Skin Bacteria Against Water Mold *Saprolegnia* spp.

**DOI:** 10.3390/jof11090649

**Published:** 2025-09-02

**Authors:** Sara Costa, Diogo Neves Proença, Artur Alves, Paula V. Morais, Isabel Lopes

**Affiliations:** 1 Centre for Environmental and Marine Studies (CESAM), Department of Biology, Campus of Santiago, University of Aveiro, 3810-193 Aveiro, Portugal; artur.alves@ua.pt (A.A.); ilopes@ua.pt (I.L.); 2 MED—Mediterranean Institute for Agriculture, Environment and Development & CHANGE—Global Change and Sustainability Institute, Faculdade de Ciências e Tecnologia, Universidade do Algarve, Campus de Gambelas, 8005-139 Faro, Portugal; daproenca@ualg.pt; 3 Centre for Mechanical Engineering, Materials and Processes (CEMMPRE), Advanced Production and Intelligent Systems (ARISE), Department of Life Sciences, University of Coimbra, Calçada Martim de Freitas, 3000-456 Coimbra, Portugal; pvmorais@ci.uc.pt

**Keywords:** skin bacteria, *Saprolegnia* spp., antagonistic effects, *Pelophylax perezi*, *S. australis*

## Abstract

Amphibian populations have experienced a severe decline over the past 40 years, driven primarily by environmental pollution, habitat destruction, climate change, and disease. This work reports, for the first time, saprolegniosis in *Pelophylax perezi* egg masses and saprolegniosis in amphibians in Portugal. After isolation and phylogenetic analysis, the pathogen was identified as *Saprolegnia australis*. Following this, the present work intended to screen a collection of *P. perezi* skin bacteria for the existence of bacterial strains with inhibitory action against the newly identified *S. australis* SC1 and two other species, *Saprolegnia diclina* SAP 1010 UE and *Saprolegnia australis* SAP 1581 UE. The results showed that various bacterial species could inhibit the growth of these three species of oomycetes. Bacteria with the most significant antagonistic action against *Saprolegnia* spp. predominantly belonged to the genus *Bacillus*, followed by *Serratia*, *Pseudomonas*, and *Aeromonas*. Despite variations in bacterial diversity among frog populations, the present study also demonstrated the presence of bacteria on frogs’ skin that were capable of inhibiting *Saprolegnia* spp., as evidenced by *in vitro* challenge assays. These findings highlight the protective function of bacteria present in amphibian skin. The observed bacterial diversity may contribute to the metabolic redundancy of the frog skin microbiome, helping to maintain its functional capacity despite shifts in the community composition. Additionally, the study found that, when providing a more advantageous environment for pathogen growth—in this case a peptone–glucose (PG) medium instead of R2A—the percentage of bacteria with moderate-to-strong antagonistic activity dropped by 13% to 4%. In conclusion, the presence of bacteria capable of inhibiting *Saprolegnia* spp. in adult individuals and across different environmental conditions may contribute to lowering the susceptibility of frog adults towards *Saprolegnia* spp., compared with that in the early stages of development, like the tadpole or egg stages.

## 1. Introduction

The genus *Saprolegnia* is a group of fungal-like organisms known as oomycetes (Oomycota, Straminipila) and includes major pathogens that cause severe losses in aquaculture [1]. They pose hazards to hosts in natural water ecosystems, including insects and crayfish, as well as amphibians, particularly in their early developmental stages [1,2,3].

In amphibians, the term ‘*Saprolegnia*-like infections’ or saprolegniosis usually refers to cotton-like patches of hyphae growing on amphibian skin or the surfaces of eggs, visible to the naked eye [2,4]. However, in most cases, no species or even genus has been characterized or identified as being the causative organism [2]. Despite this, several species of *Saprolegnia* might be involved in embryonic mortality specifically, as *S. ferax* and *S. diclina* have been associated with embryonic mortality in field amphibian populations [2,4,5].

*Saprolegnia* are saprophytic oomycetes, commonly found in aquatic environments, that can act as parasites and primary pathogens—e.g., they are capable of infecting healthy embryos of *Rana pipiens* and of *Bufo terrestris*, which can be infected by *S. ferax* and *S. diclina*, respectively [4,6]—or as opportunistic secondary pathogens, particularly in hosts that are stressed, injured, or immunocompromised [7].

These pathogen dynamics are influenced by the interaction of the host’s traits (reproductive mode, timing of egg laying, egg morphology) [6,8] and environmental factors (salinity, water quality) [9,10]. For instance, aquatic breeders with gelatinous eggs are more vulnerable than terrestrial or direct-developing species, and the timing of egg laying is important, since cold early-spring waters promote fungal growth [8]. The egg morphology, such as the jelly coat thickness or the presence of antimicrobial compounds, can either hinder or facilitate colonization [8,9]. Environmental factors are equally crucial: any factor that increases mortality or reduces development can promote fungal growth and spread—low-oxygen waters promote infection in fish, while even slight salinity can significantly boost fungal development [10,11,12].

Bacteria from the host’s skin, gut, or gill microbiota may play an important role in disease control; in fish, certain microorganisms have been reported to provide protection against *S. parasitica* infection [13]. In amphibians, it is also well established that the skin microbiome has an important and active role in defense against pathogenic agents [14,15,16]. Many studies have shown that the skin microbiome functions as an antimicrobial producer and demonstrated that bioaugmentation is an effective method to mitigate deadly diseases such as chytridiomycosis, caused by the fungus *Batrachochytrium dendrobatidis* (*Bd*) [17,18,19,20]. In addition to producing antimicrobial substances, the skin microbiota contributes to host defense by occupying ecological niches on the skin surface, thereby preventing colonization by opportunistic or pathogenic microbes [21]. This phenomenon, often described as colonization resistance, ensures that the use of the available nutrients and space is dominated by beneficial or neutral microbes, creating a competitive environment that limits pathogen establishment (e.g., bioaugmentation) [18].

In amphibians, the composition of the skin microbiome is strongly influenced by the environment itself, and the disruption of this microbial community—referred to as dysbiosis—can significantly affect the host’s susceptibility to disease [22,23,24,25].

Recently, some studies have focused on assessing the factors that may influence or disrupt the skin microbiome’s composition.

Environmental factors like temperature, rainfall, drought, or habitat disturbances (salinity, metal contamination) can reduce the *Bd*-inhibitory bacterial taxa on amphibian skin, increasing their disease susceptibility [26]. Disturbed habitats lead to altered microbial communities in amphibians, often with more variable (dysbiosis) or reduced richness [27,28]. Even amphibians’ behavior, developmental life stage, and diet drive changes in their microbiome composition [29,30,31].

However, few have investigated the existence of potential antipathogen bacteria in different environmental conditions [26,32,33]. The occurrence of bacteria known to produce antipathogen metabolites in a given environmental context is a positive predictor of host survival [34]. Nevertheless, bacterial metabolite production varies depending on factors such as temperature, nutrient availability, stress conditions, or even interactions with other community constituents [35]. To better understand bacteria–pathogen dynamics and how environmental factors could influence antimicrobial production and alter the antipathogen potential, researchers have started to explore different scenarios *in vitro*. Muletz-Wolz et al. (2017) challenged different bacteria against *Bd* strains under two temperatures and found that the inhibition potential against *Bd* was strain- and temperature-dependent [36].

In this context, the present work aimed (a) to address the existence of potential antagonistic bacteria across populations of *P. perezi* against *Saprolegnia* spp; (b) to assess whether the antagonistic bacteria’s potential is dependent on their sampling site; (c) to evaluate whether different culture conditions can influence the antagonistic capabilities of the bacteria; and (d) to identify the agent responsible for the *P. perezi* saprolegniosis infection.

## 2. Materials and Methods

### 2.1. Oomycete Isolation

A *Pelophylax perezi* (López-Seoane, 1885) egg mass with a cotton-like infection (with saprolegniosis symptoms) was found in a freshwater pond on the Aveiro University Campus (40°38′03″N/8°39′26″W). The egg mass was collected and washed with sterile distilled water (SDW), and a sample of cotton-like material was plated on a modified peptone–glucose agar medium (PG: 3 g/L peptone, 6 g/L glucose, 15g/L agar), adapted from classical formulations used for *Saprolegnia* culture [37] to reduce the nutrient load and better simulate aquatic environments.

### 2.2. Molecular Identification of Oomycetes and Phylogenetic Analysis

Molecular analysis was performed in order to identify, at the species level, the *Saprolegnia* spp. isolated from the egg mass. Genomic DNA was extracted from the mycelium, as described by Alves et al. (2008). The internal transcribed spacer (ITS) region of the ribosomal RNA gene cluster was sequenced for the isolate, as described previously [38], and deposited in GenBank (accession number MK046073). The ITS sequence of the isolate was aligned with sequences retrieved from GenBank, representing 29 ITS nrDNA reference sequences of *Saprolegnia* species according to molecular operational taxonomic units (MOTUs), previously revised by Sandoval-Sierra et al. (2014) [39].

Sequences were aligned with ClustalX [40], and maximum likelihood (ML) analyses were performed using MEGA 11 [41]. MEGA 11 was also used to determine the best-fitting DNA evolution model to be used, and the evolutionary history was inferred using the ML method and Tamura–Nei model [42]. ML analysis was performed on a neighbor-joining starting tree automatically generated by the software. The robustness of the trees was evaluated by 1000 bootstrap replications. *Phytophthora infestans* MG865512 was included as an outgroup, and the tree was visualized with TreeView [43].

### 2.3. Culture Conditions of Saprolegnia spp.

The oomycetes used in this work were the strain *Saprolegnia australis* SC1, isolated from egg masses with saprolegniosis symptoms in the freshwater pond on the Aveiro University Campus (accession number MK046073), and *S. diclina* SAP 1010 UE and *S. australis* SAP 1581 UE, from the collection of the Department of Mycology from Real Jardín Botánico CSIC, Madrid (Spain). Isolates were maintained in PG agar, and the pure cultures were cryopreserved at −80 °C in 20% (*v*/*v*) glycerol.

### 2.4. Bacterial Strains

To screen bacteria with an antagonistic effect against *Saprolegnia* spp., a collection of 196 bacterial isolates was used (Table S1). These isolates were previously isolated from the skin microbiomes of *P. perezi* frogs [25,44]. They were sourced from populations inhabiting various environmental conditions, such as freshwater, brackish water and metal-contaminated ecosystems, during previous studies that evaluated and compared the microbial diversity of populations of *P. perezi* from different environmental conditions [25,44]. The results showed significant differences in bacterial composition between populations, particularly those influenced by environmental constraints like increased salinity or influenced by anthropogenic activities like mining.

Bacterial isolates were grown on Reasoner’s 2A (R2A) agar at 23 °C for 5 days before being evaluated for antagonistic activity against oomycetes from *Saprolegnia* spp.

### 2.5. Screening of Antagonistic Bacteria

**Figure 1:**
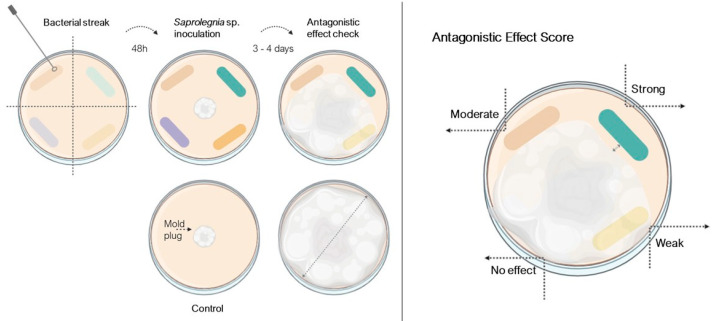
Illustration of the experimental setup and scoring method for the evaluation of the antagonistic activity of bacterial isolates against *Saprolegnia* spp. on agar plates. **Left panel**: Experimental workflow—streaking selected bacterial strains in radial lines on an agar plate and allowing bacterial growth. A *Saprolegnia* sp. mycelial plug is then placed at the center of the plate. After co-incubation, plates are examined for zones of inhibition, indicating antagonistic effects. A control plate containing only the mold plug is included for comparison. **Right panel**: Antagonistic effect is visually scored based on the extent of fungal growth inhibition surrounding each bacterial streak. The scale ranges from “no effect” to “strong” inhibition, with larger clear zones indicating higher antagonistic activity. Color-coded bacterial streaks in both panels represent different bacterial strains with varying levels of inhibitory activity.

The 196 bacterial isolates were streaked as thick bands on opposite edges of the R2A plates and allowed to grow for 48 h before inoculation with *Saprolegnia* spp. (Figure 1). Then, a 4 mm diameter disc of the oomycete was cut from an actively growing culture and placed in the center of the R2A plate. The procedure was replicated for each *Saprolegnia* strain. The Petri dishes were incubated at 23 °C in the dark for 3 to 4 days, or until the mycelium of *Saprolegnia* spp. covered the entire plate in the control. The antagonistic effect shown by the bacteria was scored as follows: (a) strong (there was an inhibition halo), (b) moderate (mold did not grow on top of the bacteria), (c) weak (mold growth on top but with thin and reduced mycelia), and (d) no effect (*Saprolegnia* spp. growth with normal macroscopic aspects, similar to the control).

Those bacterial strains that showed strong and moderate antagonistic activity in the initial screening on R2A against at least one of the *Saprolegnia* strains were then challenged again against all *Saprolegnia* spp. in PG agar, to understand whether they maintained this antagonistic activity in a different growth medium with higher nutrient loading compared with R2A.

## 3. Results

### 3.1. Molecular Identification of Oomycetes

**Figure 2:**
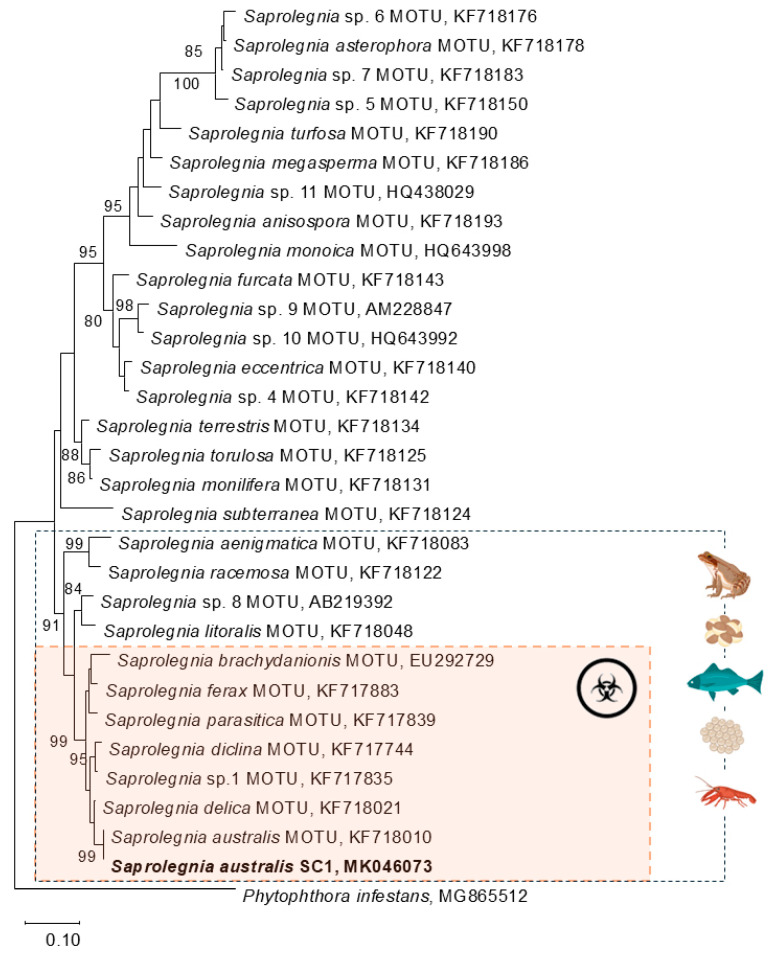
Phylogenetic dendrogram based on a comparison of the ITS nrDNA gene sequence of strain *Saprolegnia australis* SC1 and other MOTUs of the genus *Saprolegnia* obtained from clustering optimization analysis [39]. The tree was created using the maximum likelihood method. The numbers on the tree indicate the percentages of bootstrap sampling, derived from 1000 replications; values below 70% are not shown. The isolate characterized in this study is indicated in bold. Strain *Phytophthora infestans* MG865512 was used as an outgroup to root the tree. Scale bar, 1 inferred nucleotide substitution per 10 nt. The shaded clade (in orange) includes taxa with recognized pathogenic potential, such as *S. parasitica*, *S. ferax*, and *S. diclina*, which are frequently associated with infections in fish, amphibians, and crustaceans, as indicated by the icons on the right. Other clades consist mainly of species considered to be saprophytic.

The phylogenetic relationships of the *Saprolegnia* SC1 isolate obtained in this study with other members of the genus *Saprolegnia* were elucidated using ITS sequences (Figure 2). The ITS sequence of the isolate was aligned with sequences retrieved from GenBank, representing 29 ITS reference sequences of *Saprolegnia* species according to molecular operational taxonomic units (MOTUs) defined by Sandoval-Sierra et al. (2014) [39]. The isolate obtained in this study clustered within a strongly supported clade (99% bootstrap) that included species pathogenic to both fish and amphibians: *S. ferax*, *S. parasitica*, *S. diclina*, *S. delica* and *S. brachydanionis*. Within this clade, isolate *Saprolegnia* SC1 clustered with the sequence of *Saprolegnia australis*, forming a sub-clade with high bootstrap support (99%).

### 3.2. Bacterial Screening

A total of 196 bacterial isolates were challenged against the three strains of *Saprolegnia* spp. (see details in Table 1, Tables S1 and S2).

Among the three *Saprolegnia* strains tested, the isolate identified in this study—*S. australis* SC1—was inhibited (weakly, moderately, or strongly) by the highest number of bacterial isolates (68), followed by *S. diclina* SAP 1010 UE (49) and *S. australis* SAP 1581 UE (44) (Table 1). It was also the isolate for which a lower number of bacterial isolates induced no effect on growth (128), compared to *S. diclina* SAP 1010 UE (147) and *S. australis* SAP 1581 UE (152).

**Figure 3:**
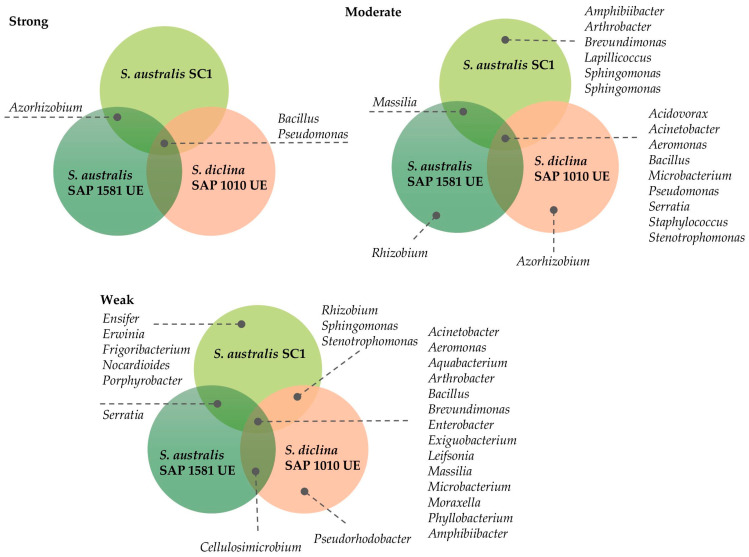
Venn diagrams representing shared genera with inhibitory capacities (strong, moderate, and weak) tested against the three *Saprolegnia* spp.

The bacterial inhibition of *Saprolegnia* varied in strength and consistency across isolates (Figure 3). A small subset of taxa, particularly *Bacillus* and *Pseudomonas*, displayed strong antagonistic activity and were shared across isolates, indicating their broad inhibitory potential. Moderate antagonists were more taxonomically diverse, including genera such as *Aeromonas*, *Serratia*, and *Stenotrophomonas*, but their effects were less consistent among isolates. Weak antagonists encompassed the largest number of genera, many of which were shared across isolates, although their inhibitory effects were limited. Overall, these results suggest that, while numerous bacterial taxa can interact with *Saprolegnia*, only a few consistently exert strong suppression, highlighting the importance of the microbial community composition in shaping pathogen dynamics.

According to the environmental sampling site characteristics, 106 (54%) strains were isolated from freshwater amphibian populations, 22 (11%) strains from brackish water populations, and 68 strains (35%) from metal-contaminated populations.

**Figure 4:**
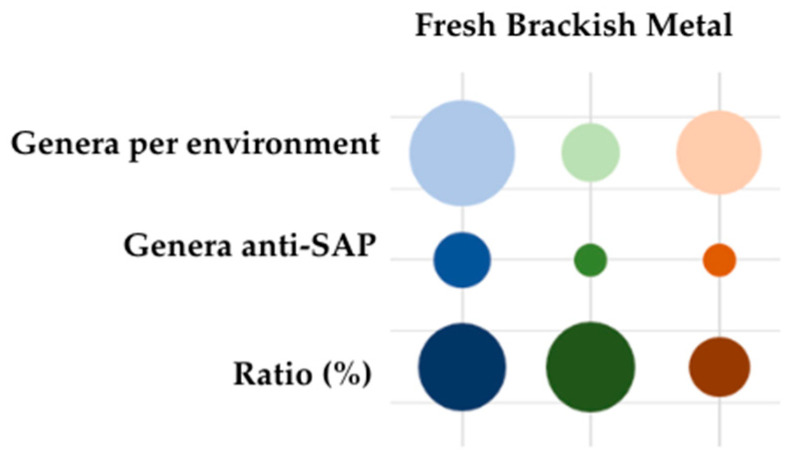
Ratio representation of the proportion of the total genera tested in this work, by sampling environment, in comparison with the total genera with antagonistic effects against *Saprolegnia* spp.

Analyzing the frog populations sampled (Table 1; Figure 4), the results indicate that all populations have bacterial isolates with antagonistic potential against *Saprolegnia* spp. On average, the population hosts between 21% and 35% of bacteria, depending on the *Saprolegnia* isolate tested, showing anti-*Saprolegnia* potential (anti-SAP) ranging from weak to strong. In general, frog populations in brackish and metal-contaminated environments exhibited the highest percentages of bacterial isolates effective against the three *Saprolegnia* isolates.

Despite the results indicating that freshwater environments have a higher number of bacterial isolates with anti-SAP activity, the percentage is inverted when considering the number of isolates tested per environment (Figure 4).

Relative to the proportion of the number of genera represented in each environmental condition, freshwater and brackish water environments contain around 30% of the genera with potential antagonistic activity against *Saprolegnia* spp. growth (anti-SAP). In contrast, the metal-contaminated samples only comprise 15% of such genera. This indicates that metal-contaminated environments have a lower relative diversity of genera with anti-SAP potential, compared with freshwater and brackish water environments.

**Figure 5:**
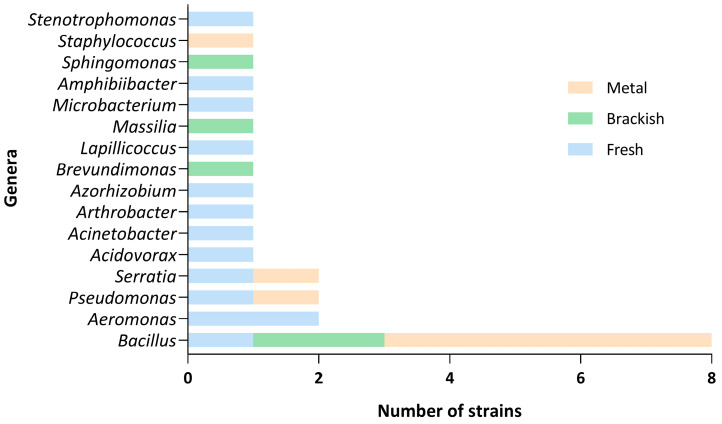
Number of bacterial strains, per 16 genera, with antagonistic activity against at least one of the tested strains of *Saprolegnia*. The other 45 bacterial genera isolated from *Pelophylax perezi* populations according to the sampling site (metal contaminated, brackish, and freshwater) are not represented.

When analyzing the results in terms of the 61 genera tested, only 16 had some antagonistic effect over the *Saprolegnia* strains (Figure 5, Supplementary Table S2). The most representative genus, with strains from all three types of habitats, was *Bacillus*, with half (eight) of the strains tested having the potential to inhibit *Saprolegnia* spp. growth. Strains of *Serratia*, *Pseudomonas*, and *Aeromonas* were the following taxonomic groups that showed effects against this pathogen.

In a second approach, 26 bacterial strains, which previously showed activity against at least one of the *Saprolegnia* spp., were tested again in different conditions to compare the effects of different media in their capacity to maintain antagonistic activity against *Saprolegnia* spp.

*Saprolegnia* spp. strains were co-cultured with bacteria in two different media to assess how nutrient availability influences their interactions. The R2A medium, representing a nutrient-poor environment typical of aquatic habitats, is less favorable for *Saprolegnia* but ideal for isolating amphibian skin bacteria. Conversely, the nutrient-rich PG medium supports rapid microbial growth. This environment may either enhance *Saprolegnia* proliferation or trigger the bacterial production of inhibitory metabolites, although bacteria might instead focus on growth over secondary metabolism.

**Figure 6:**
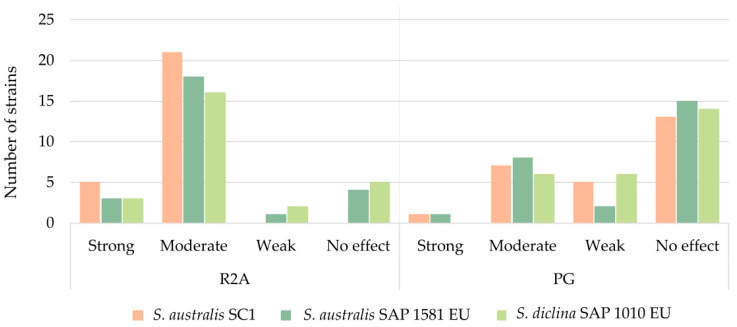
Number of bacterial strains with different antagonistic capacities against *Saprolegnia* spp. after exposure to different culture media (R2A and PG).

Accordingly, when we challenged the mold and the bacterial strains in PG agar medium, the number of bacterial strains with activity against *Saprolegnia* spp. decreased (Figure 6, Supplementary Table S2). From the total of 26 strains, on average, while, in R2A, 14% showed strong inhibition, 71% moderate, 4% weak, and 12% showed no effect against *Saprolegnia* spp., in PG, 3% showed strong inhibition, 27% moderate, 17% weak, and 54% showed no effect.

In R2A, *S. diclina* SAP 1010 EU was the strain for which fewer bacterial isolates exerted an antagonistic effect of any intensity; however, it was very close to *S. australis* SAP 1581 EU and *S. australis* AV. In PG, as mentioned above, the percentage of strains with no effect was higher than in R2A; however, *S. australis* AV was the strain that the most bacterial strains were able to inhibit.

## 4. Discussion

### 4.1. Bacteria’s Antagonistic Growth Effects Against Saprolegnia spp.

In relation to the present work, the three strains of *Saprolegnia* spp. were challenged against 196 bacterial isolates. Only 26 isolates were able to affect the growth or mycelial appearance of at least one strain of *Saprolegnia*. According to the obtained results, *Bacillus*, *Pseudomonas*, and *Serratia* were the genera with the highest number of isolates, inducing an antagonistic growth effect on *Saprolegnia* spp. The species *Bacillus arybhattai*, *B. aerophilus*, *B. safensis*, *Pseudomonas meridiana*, *Serratia nematodiphila*, and *Azorhizobium doebereinerae* were shown to be effective candidates as anti-SAP agents in the *in vitro* assays, exhibiting strong inhibition of the growth of the three species.

The bacterial genera identified in this study, including *Bacillus*, *Pseudomonas*, *Serratia*, and others isolated from the skin of frogs, are consistent with previous reports demonstrating inhibitory activity against *Saprolegnia* spp. in various hosts, including fish and amphibians, as well as against other pathogenic microorganisms. For example, in fish, studies have shown that some bacterial strains can inhibit the growth of *Saprolegnia* spp. *in vitro*, including mainly species from the genera *Pseudomonas (P. fluorescens*, *P. alcaligenes*, *P. saccharophila*, *P. aeruginosa*), *Aeromonas* (*A. caviae*, *A. eucrenophila*, *A. media*, *A. sobria)*, *Serratia (S. marcescens*, *S. fonticola)*, *Bacillus*, and *Yersinia kristensenii*, among others [13].

In fact, microbial symbionts on vertebrate skin play a crucial role in protecting the host against pathogens. Most of the works published so far have focused on symbiotic bacteria that produce antifungal metabolites that inhibit *B. dendrobatidis* (*Bd*) infections [18,19]. For example, the bacterium *Janthinobacterium lividum* produces the metabolites violacein and indole-3-carboxaldehyde (I3C), which are capable of inhibiting *Bd* growth. This bacterial strain was isolated in several amphibian species, like red-backed salamanders (*Plethodon cinereus*) and mountain yellow-legged frogs (*Rana muscosa*) [19,45]. Other antifungal compounds found in the amphibian microbiomes of *R. muscosa* and *P. cinereus* include 2,4-diacetylphloroglucinol (2,4-DAPG), a metabolite produced by both *Pseudomonas* *fluorescens* and *Lysobacter gummosus* [46,47]; indole-3-ethanol (tryptophol), produced by *P. fluorescens* [48]; and prodigiosin, found in several species of *Serratia* [49]. *Bd-*inhibitory bacterial isolates are widely distributed among phyla, although some genera, such as *Stenotrophomonas*, *Aeromonas*, and *Pseudomonas*, have large proportions of inhibitory isolates [50].

Other works have conducted *in vitro* investigations of antimicrobial-producing bacteria against other pathogenic agents, such as *Aeromonas salmonicida* [32,33]. Coelho et al. (2022) searched for bacterial antimicrobial activity against *A. salmonicida* on the Iberian frog (*Rana iberica*) skin microbiome [32]. Among the strains that they genetically identified, they found that the most common genera with antimicrobial activity were *Pseudomonas*, *Chryseobacterium*, and *Stenotrophomonas*. Moreover, Afonso et al. (2022) identified that, for the fire salamander (*Salamandra salamandra*), the most common bacterial genera present in its skin microbiome with antimicrobial activity were *Pseudomonas*, *Kluyvera*, and *Flavobacterium* [33]. However, both authors only identified a small percentage of the isolates that presented antimicrobial activity against *A. salmonicida*.

### 4.2. Origins of the Isolate Populations and Environmental Conditions

Although saprolegniosis mainly affects the early life stages of amphibians, it may appear in adult individuals [2]. The bacterial isolates tested in the present work were sampled in adult frogs of *P. perezi* from different field populations inhabiting ponds with different environmental conditions. Focusing on environmental influences on the skin microbiome composition and function, published works reveal that the microbiome is deeply influenced by environmental characteristics [25]. It is logical to posit that animals will be colonized by organisms existing in the surrounding environment, selected based on host–microbiome interactions, which will be influenced by the ecology of the animal (e.g., if it is exclusively aquatic or if it spends the majority of its time inland or in water) and by the characteristics of the mucous layer and the antimicrobial peptides produced by the bacterium itself. Assis et al. (2017) studied the microbiome composition between sympatric species of amphibians from continuous and fragmented forests [51]. In the four species studied, only *Proceratophrys boiei* showed significant differences between habitat typologies. In addition, bacterial strains with pathogen-inhibitory potential were found in all species studied, regardless of the habitat typology. Albecker et al. (2019) compared populations of *Hyla cinerea* from coastal and inland habitats (with a salinity influence) and concluded that the skin microbial composition was different [52]. However, they did not find differences in the mean species richness of the skin microbial communities or in the proportions of the three bacterial taxa with potential anti-*Bd* properties *(J. lividum*, *Pseudomonas* spp., and *S. marcescens*).

In the present work, bacteria collected from the skin of *Pelophylax perezi* inhabiting three different environments—non-contaminated freshwater ponds, ponds with increased salinity, and ponds contaminated with metals—were analyzed for their influences on the growth of three *Saprolegnia* isolates. The core cultivable microbiota found in the three environments was composed of different species of the genera *Bacillus*, *Brevundimonas*, *Rhizobium*, and *Sphingomonas* [25,44]. Therefore, despite the results showing that the freshwater environment had a higher number of bacterial isolates with anti-SAP activity (Table 1), it was also the environment with the highest number of isolates tested. In proportion to the number of isolates tested per environment, the percentage was inverted. Brackish and metal environments presented the highest percentages of bacterial isolates with antagonistic activity against the *Saprolegnia* isolates, and this could be explained by the fact that harsh or dynamic environments, like contaminated sites, tend to be hotspots for microbial evolution and drive the emergence of metabolically versatile and competitive strains (e.g., by the production of secondary metabolites and antibiotic resistance) [53].

The microbial community diversity in environments affected by stressors is often reduced in overall richness but shifted towards specialized or resistant organisms, because these communities are shaped by strong selection pressures favoring survival traits over taxonomic variety.

The environment with the highest diversity of genera represented was the freshwater non-contaminated environment (42 genera), followed by the metal-contaminated one (27) and then the brackish water (13) environment. In the present study, it was found that all sampled populations had bacterial isolates with antagonistic potential against *Saprolegnia* spp. (anti-SAP). Beyond the sampling site, when we analyzed the environmental conditions of the population, we also found anti-SAP isolates in both freshwater and brackish water and in metal-contaminated populations.

Freshwater and brackish water populations, however, showed 30% of the genera isolated from each population with anti-SAP activity, and metal contaminated populations showed only 15%. These results suggest that metal contamination may reduce not only the overall diversity but also the functional capacity (i.e., antagonistic potential) of microbial communities against *Saprolegnia* spp., and environments with higher microbial diversity may offer more robust natural protection against pathogens like *Saprolegnia*.

These findings allow us to posit that microbial communities will undergo restructuring according to the bacterial pool available in the environment, but they will not necessarily lose their basal function of primary protection by competing with other potential colonizers. The wide distribution of this trait suggests that functional redundancy occurs in these bacterial communities and may be indicative of the key role of these skin symbiont communities in host protection, especially in the face of fungal pathogens. In addition, non-contaminated freshwater environments support both high diversity and high functional potential in terms of antagonistic activity and contamination (e.g., with metals), and environmental stress (e.g., salinity) negatively affects microbial diversity and reduces the proportion of beneficial (anti-SAP) bacteria.

### 4.3. Substrate Impacts on the Antagonistic Effects of Bacteria Against Saprolegnia spp.

The *Saprolegnia* spp. strains were challenged with bacteria in two different culture media in order to understand whether variations in nutrient composition could affect their interactions. The R2A medium was used to simulate a nutrient-poor environment (e.g., aquatic), which is less favorable to *Saprolegnia* spp. and also the most indicated for isolation and work with amphibian skin bacteria [54]. In such nutrient-limited conditions, bacteria and pathogens may compete for scarce resources, often resulting in antagonistic interactions. Bacteria may gain an advantage by producing antimicrobial compounds [55]. On the other hand, the PG medium is rich in organic nitrogen and simple sugars, which makes it suitable for supporting robust, fast-growing microorganisms, and it was used to simulate nutrient-rich environments, which can have dual effects. In this case, two alternative responses could occur: it may promote *Saprolegnia* growth by providing ample resources for reproduction and invasion; alternatively, it could stimulate bacteria to either increase the production of secondary metabolites that inhibit fungal growth or prioritize their own growth over secondary metabolite production. This approach aimed to elucidate how environmental conditions modulate the effects of bacteria on the growth and development of *Saprolegnia* spp.

For example, *J. lividum* produces varying levels of violacein, an antifungal pigment, depending on the culture medium used. Specifically, violacein production is often reduced when *J. lividum* is grown in rich media like Luria–Bertani (LB) broth, compared to minimal media such as Davis Minimal Broth with glycerol (DMBgly) [56].

In the present work, 196 bacterial strains were challenged with three *Saprolegnia* spp. strains in R2A, resulting in 26 isolates with the strong to moderate inhibition of at least one of the *Saprolegnia* isolates. Then, these 26 isolates were challenged again, but in PG culture media, and only 35%—which corresponded to nine strains—maintained their antagonistic activity against the molds.

The results also highlight that the strains that maintained strong to moderate antagonistic activity against *Saprolegnia* spp. in PG were mostly isolated from non-contaminated freshwater environments.

The results of the present work reveal that the type of culture medium significantly affects the interaction of the pathogen *Saprolegnia* spp. and bacteria *in vitro*, likely due to alterations in nutrient availability, which influence microbial competition and physiological responses.

### 4.4. Pathogen Identification as Saprolegnia australis

To our knowledge, this is the first time that *Saprolegnia* infections in amphibians have been reported in Portugal and in the common green frog species *Pelophylax perezi*. The first published study of amphibians with *S. australis* infection was performed by Kim et al. (2008), who identified tadpoles of the gold-spotted pond frog, *Rana plancyi chosenica*, with symptoms of saprolegniosis in Korea and genetically identified the species as *Saprolegnia australis* [57]. In fact, like other species of water molds, *S. australis* has been isolated from freshwater worldwide, showing the ubiquity of these organisms [39].

*Saprolegnia* species are known to be frequently observed and isolated in fish farms, namely affecting salmonid aquaculture [58]. Previous works found that *S. australis* was significantly associated with samples from the embryonic stages of salmonids [58,59]. However, this association was not found for adult stages of fish. In line with these findings, this *Saprolegnia* species has been isolated in other farms in adult fish but has not been shown to significantly affect individuals compared with other species like *S. parasitica* [37]. *Saprolegnia australis* was consistently isolated from the lesions of affected fish, solely or combined with other pathogens like *Aeromonas hydrophila* [60].

In addition, this species is associated with other groups of organisms, such as crustaceans. It is one of the pathogens responsible for crayfish plague, which causes high mortality and ulcerative outbreaks in crayfish. Several studies have reported *S. australis* affecting native and invasive species, such as *Austropotamobius pallipes* in Spain [61], *Astacus astacus* and *Orconectes limosus* in Czech Republic outbreaks [61], *O. limosus* in Germany [62], and *O. propinquus* in the USA [63].

The non-specificity of the host suggests that fish or crayfish are potential carriers of pathogens of other organisms, such as amphibians, as suggested by other published works for other *Saprolegnia* species [64].

Overall, *Saprolegnia* appears to be a generalist in terms of host range. Phylogenetic analysis shows the clear clustering of species into distinct lineages with strong bootstrap support. A well-defined clade (highlighted in orange, Figure 2) includes pathogenic species such as *S. parasitica*, *S. ferax*, *S. diclina*, and *S. australis*, all strongly associated with infections in fish, amphibians, and crustaceans. This clade represents taxa with recognized pathogenic potential in both aquaculture and natural ecosystems [1]. In contrast, other *Saprolegnia* species (upper part of the tree) form separate lineages that are less frequently linked to disease and likely play a primarily saprophytic role in aquatic environments [65,66]. Host-specific associations, indicated by icons, suggest that pathogenic species tend to exhibit a broader host range compared to non-pathogenic or opportunistic taxa, as mentioned above.

The correlation between phylogenetic placement and pathogenic behavior supports the idea that virulence traits are conserved within certain evolutionary lineages of oomycetes. These findings emphasize the value of phylogenetic frameworks in predicting pathogenic potential and improving our understanding of host–pathogen dynamics in aquatic ecosystems.

## 5. Conclusions

The choice of culture medium significantly influenced the ability of bacteria to inhibit *Saprolegnia* spp. In nutrient-limited media, like R2A, which better simulates natural environments, bacterial isolates exhibited enhanced antifungal activity, probably due to the increased production of secondary metabolites as a stress response. Conversely, high-nutrient media, like PG, promote rapid bacterial growth, probably leading to the reduced production of inhibitory compounds and contributing to its diminished antagonistic effects. These findings highlight the importance of selecting appropriate culture conditions when studying microbial interactions and antifungal potential, as the medium composition can impact both pathogen growth and the efficacy of bacterial antagonism.

The next step should be to test co-cultures instead of monocultures against the mold. Upon examining bacterial isolates from the skin microbiome, it is expected that co-cultures of bacterial species or strains will result in the greater inhibition of *Saprolegnia* spp. than in monocultures. Understanding the mechanisms underlying the inhibition of water mold growth by bacteria could facilitate applications in nature to halt amphibian population declines.

The present work allowed the identification of *S. australis* as a potential pathogen associated with *Pelophylax perezi* eggs in the wild environment. In addition, the results show the existence of bacteria in the skin of *P. perezi* capable of inhibiting water molds like *Saprolegnia* spp. However, saprolegniosis is a disease associated with the early developmental stages, and no studies have been conducted in order to evaluate the existence of this type of inhibitory bacteria in these development stages, mainly in egg masses. According to the results of this work, the presence of these bacteria in adult individuals could contribute to lower susceptibility towards *Saprolegnia* spp., compared with the early stages of development, like tadpoles or eggs.

## Figures and Tables

**Table 1:** Summary table of the number of strains per response, per *Saprolegnia* spp. count of isolates challenged against the three strains of *Saprolegnia* spp. In parentheses, the percentage of bacteria relative to the total isolates corresponding to the environment of each isolation site is shown. All challenge assays were performed in R2A agar medium.

*Saprolegnia* Species	Type of Bacterial Antagonistic Response	Number of Strains Per Response	Ratio of Bacterial Strains According to Isolation Site Environment (%)		
			Fresh	Brackish	Metal
*S. australis* SC1	Strong	5 (3%)	1.9	4.5	2.9
	Moderate	20 (10%)	9.4	18.2	8.8
	Weak	43 (22%)	20.8	22.7	23.5
	No effect	128 (65%)	67.9	54.5	64.7
*S. australis* SAP 1581 UE	Strong	3 (2%)	1.9	4.5	0.0
	Moderate	20 (10%)	8.5	9.1	13.2
	Weak	21 (11%)	9.4	13.6	11.8
	No effect	152 (78%)	80.2	72.7	75.0
*S. diclina* SAP 1010 UE	Strong	3 (2%)	0.9	9.1	0.0
	Moderate	17 (9%)	8.5	0.0	11.8
	Weak	29 (15%)	12.3	31.8	13.2
	No effect	147 (75%)	78.3	59.1	75.0

## Supplementary Materials

The following supporting information can be downloaded at https://www.mdpi.com/article/10.3390/jof11090649/s1. Table S1: Total bacterial isolates tested against *Saprolegnia* species; Table S2: Bacterial isolates with an antagonistic effect against *Saprolegnia* spp., comparing activity between R2A and PG.
