# Supplementary material for: Potential Protective Role of Amphibian Skin Bacteria Against Water Mold Saprolegnia spp
Source: J Fungi (Basel). 2025 Sep 2;11(9):649. doi: 10.3390/jof11090649 (PMC12471046; doi:10.3390/jof11090649)
Supplement: Supplementary file 1 [file jof-11-00649-s001.zip › S1 Bacterial list.pdf]

Table S1- Total Bacterial isolates tested against *Saprolegnia* species. SL-Salreu; LB-Lagoa das Braças; LC-Lagoa do Cão; BR1-Barragem de Reguengos 1; BR2- Barragem de Reguengos 2; TP/ AF- Água Forte.

#	closest EzTaxon (nº)	Genera	CODE	Sampling site	Environment	*S. australis* AV	*S. australis* SAP	*S. diclina* SAP	GenBank
1	*Bacillus arybhattai* B8W22	*Bacillus*	SL12_5	SL	Brackish	strong	strong	strong	KT720429
2	*Bacillus vietnamensis* 15-1	*Bacillus*	SL12_1	SL	Brackish	weak	weak	weak	KT720427
3	*Bacillus aerophilus* 28K	*Bacillus*	SL12_7	SL	Brackish	moderate	moderate	strong	KT720430
4	*Amphibiibacter pelophylacis*	*Amphibiibacter*	SL12_8	SL	Brackish	-	-	-	KT720394
5	*Moraxella osloensis* AerLab-37	*Moraxella*	SL12_9	SL	Brackish	weak	weak	weak	KT720395
6	*Pseudorhodobacter ferrugineus* IAM 12616	*Pseudorhodobacter*	SL12_6	SL	Brackish	-	-	weak	KT720393
7	*Sphingomonas faeni* MA-olki	*Sphingomonas*	SL12_3	SL	Brackish	weak	-	-	KT720392
8	*Paenibacillus pabuli* JCM 9074	*Paenibacillus*	SL12_2	SL	Brackish	-	-	-	KT720428
9	*Methylobacterium marchantiae* JT1	*Methylobacterium*	SL2_1	SL	Brackish	-	-	-	KT720396
10	*Bacillus mycoides* ATCC 6462	*Bacillus*	SL2_2	SL	Brackish	-	-	weak	KT720431
11	*Micrococcus antarcticus* T2	*Micrococcus*	SL2_4	SL	Brackish	-	-	-	KT720432
12	*Paracoccus marinus* KKL-A5	*Paracoccus*	SL2_5	SL	Brackish	-	-	-	KT720397
13	*Deinococcus grandis* DSM 3963	*Deinococcus*	SL2_6	SL	Brackish	-	-	-	KT720433
14	*Flavobacterium tegetincola* ACAM 602	*Flavobacterium*	SL2_8	SL	Brackish	-	-	-	KT720399
15	*Massilia aurea* AP13	*Massilia*	SL2_9	SL	Brackish	moderate	moderate	weak	KT720400
16	*Rhizobium rosettiformans* W3	*Rhizobium*	SL2_7	SL	Brackish	-	-	-	KT720398
17	*Rhodopseudomonas faecalis*	*Rhodopseudomonas*	SL3_11	SL	Brackish	-	-	-	KT720402
18	*Arthrobacter oxydans* DSM 20119	*Arthrobacter*	SL3_1	SL	Brackish	weak	-	weak	KT720434
19	*Brevundimonas bullata* IAM 13153	*Brevundimonas*	SL3_9	SL	Brackish	moderate	-	-	KT720405
20	*Microvirga zambiensis WSM3693(T)*	*Microvirga*	SL3_10	SL	Brackish	-	-	-	KT720401
21	*Massilia aerilata 5516S-11(T)*	*Massilia*	SL3_5	SL	Brackish	weak	weak	weak	KT720403
22	*Sphingomonas glacialis C16y(T)*	*Sphingomonas*	SL3_8	SL	Brackish	moderate	-	-	KT720404
23	*Frigoribacterium faeni 801(T)*	*Frigoribacterium*	LB13_1	LB	Freshwater	weak	-	-	KT720416
24	*Aquabacterium parvum B6(T)*	*Aquabacterium*	LB13_2	LB	Freshwater	weak	weak	weak	KT720382
25	*Frigoribacterium faeni 801(T)*	*Frigoribacterium*	LB13_4	LB	Freshwater	weak	-	-	KT720417
26	*Azorhizobium doebereinerae*	*Azorhizobium*	LB13_5	LB	Freshwater	strong	strong	moderate	KT720376
27	*Piscinibacter aquaticus IMCC1728 (T)*	*Piscinibacter*	LB13_6	LB	Freshwater	weak	weak	weak	KT720383
28	*Staphylococcus warneri ATCC 27836 (T)*	*Staphylococcus*	LB13_7	LB	Freshwater	-	-	-	KT720418
29	*Rhizobium rosettiformans W3(T)*	*Rhizobium*	LB13_10	LB	Freshwater	weak	-	weak	KT720380
30	*Azohydromonas lata IAM 12599(T)*	*Azohydromonas*	LB13_11	LB	Freshwater	-	-	-	KT720381
31	*Pelomonas aquatica CCUG52575(T)*	*Pelomonas*	LB13_11	LB	Freshwater	-	-	-	-
32	*Geodermatophilus obscurus DSM 43160(T)*	*Geodermatophilus*	LB7_1	LB	Freshwater	-	-	-	KT720421
33	*Nocardioides alpinus Cr7-14(T)*	*Nocardioides*	LB7_2	LB	Freshwater	weak	-	-	KT720422

34	*Microbacterium lacus A5E-52(T)*	*Microbacterium*	LB7_3	LB	Freshwater	weak	weak	weak	KT720423
35	*Microbacterium lacus A5E-52(T)*	*Microbacterium*	LB7_4	LB	Freshwater	weak	-	-	KT720424
36	*Lapillicoccus jejuensis*	*Lapillicoccus*	LB7_6	LB	Freshwater	moderate	-	-	KT720425
37	*Brevundimonas nasdae GTC 1043(T)*	*Brevundimonas*	LB7_8	LB	Freshwater	weak	-	-	KT720390
38	*Microbacterium lacus A5E-52(T)*	*Microbacterium*	LB7_9	LB	Freshwater	weak	-	-	KT720426
39	*Bradyrhizobium liaoningense 2281*	*Bradyrhizobium*	LB7_11	LB	Freshwater	-	-	-	KT720388
40	*E-iguobacterium undae DSM 14481(T)*	*Exiguobacterium*	LB1_1	LB	Freshwater	weak	-	-	KT720415
41	*Acinetobacter beijerinckii 58a(T)*	*Acinetobacter*	LB1_3	LB	Freshwater	-	-	-	KT720379
42	*Bosea lathyri LMG26379(T)*	*Bosea*	LB1_4	LB	Freshwater	-	-	-	KT720384
43	*Microbacterium testaceum DSM 20166(T)*	*Microbacterium*	LB1_5	LB	Freshwater	weak	-	-	KT720419
44	*Sphingomonas faeni MA-olki(T)*	*Sphingomonas*	LB1_6	LB	Freshwater	weak	-	weak	KT720385
45	*Amphibiibacter pelophylacis*	*Amphibiibacter*	LB1_7	LB	Freshwater	moderate	-	-	KT720386
46	*Nocardioides furvisabuli SBS-26(T)*	*Nocardioides*	LB1_8	LB	Freshwater	-	-	-	KT720420
47	*Roseomonas stagni HS-69(T)*	*Roseomonas*	LB1_9	LB	Freshwater	-	-	-	KT720387
48	*Erwinia toletana A37(T)*	*Erwinia*	LB1_10	LB	Freshwater	-	-	-	KT720377
49	*Porphyrobacter tepidarius DSM 10594(T)*	*Porphyrobacter*	LB1_11	LB	Freshwater	weak	-	-	KT720378
50	*Pseudomonas meridiana*	*Pseudomonas*	9F11	LC	Freshwater	strong	strong	strong	KY611767
51	*Pseudomonas reinekei*	*Pseudomonas*	9F12	LC	Freshwater	-	-	-	KY611768
52	*Pseudomonas helmanticensis*	*Pseudomonas*	9F13	LC	Freshwater	-	-	-	KY611769
53	*Acinetobacter johnsonii*	*Acinetobacter*	9F15	LC	Freshwater	-	-	-	KY611770
54	*Acinetobacter junii*	*Acinetobacter*	9F2	LC	Freshwater	-	-	-	KY611772
55	*Microbacterium oxydans*	*Microbacterium*	9F3	LC	Freshwater	-	-	-	KY611773
56	*Acinetobacter johnsonii*	*Acinetobacter*	9F5	LC	Freshwater	-	-	-	KY611775
57	*Pseudomonas mucidolens*	*Pseudomonas*	9F6	LC	Freshwater	-	-	-	KY611776
58	*Acinetobacter indicus*	*Acinetobacter*	9F7	LC	Freshwater	-	-	-	KY611777
59	*Acinetobacter oryzae*	*Acinetobacter*	10M1	LC	Freshwater	-	-	-	KY611614
60	*Acinetobacter oryzae*	*Acinetobacter*	10M3	LC	Freshwater	-	-	-	KY611617
61	*Enterobacter asburiae*	*Enterobacter*	10M4	LC	Freshwater	-	-	-	KY611618
62	*Acinetobacter guillouiae*	*Acinetobacter*	10M6A	LC	Freshwater	moderate	moderate	moderate	KY611620
63	*Enterobacter asburiae*	*Enterobacter*	10M7A	LC	Freshwater	-	-	-	KY611622
64	*Acinetobacter johnsonii*	*Acinetobacter*	11M3A	LC	Freshwater	-	-	-	KY611626
65	*Acinetobacter oryzae*	*Acinetobacter*	11M4A	LC	Freshwater	-	-	-	KY611629
66	*Serratia fonticola*	*Serratia*	11M5	LC	Freshwater	moderate	moderate	moderate	KY611630
67	*Acinetobacter beijerinckii*	*Acinetobacter*	11M6	LC	Freshwater	-	-	-	KY611631
68	*Acinetobacter johnsonii*	*Acinetobacter*	11M7	LC	Freshwater	weak	weak	weak	KY611632
69	*Aeromonas punctata subsp. punctata*	*Aeromonas*	11M8B	LC	Freshwater	moderate	moderate	weak	KY611633
70	*Arthrobacter niigatensis*	*Arthrobacter*	12F1	LC	Freshwater	moderate	weak	-	KY611635

71	*Curtobacterium flaccumfaciens*	*Curtobacterium*	12F10	LC	Freshwater	-	-	-	KY611636
72	*Arthrobacter chlorophenolicus*	*Arthrobacter*	12F11A	LC	Freshwater	weak	-	-	KY611637
73	*Cellulosimicrobium terreum*	*Cellulosimicrobium*	12F11Bb	LC	Freshwater	-	weak	weak	KY611639
74	*E-iguobacterium acetylicum*	*Exiguobacterium*	12F11Ca1	LC	Freshwater	-	weak	weak	KY611640
75	*Citrobacter freundii*	*Citrobacter*	12F12	LC	Freshwater	-	-	-	KY611643
76	*Arthrobacter arilaitensis*	*Arthrobacter*	12F13	LC	Freshwater	-	-	-	KY611644
77	*Acinetobacter beijerinckii*	*Acinetobacter*	12F14	LC	Freshwater	-	-	-	KY611645
78	*Aeromonas punctata subsp. punctata*	*Aeromonas*	12F16	LC	Freshwater	weak	weak	weak	KY611646
79	*Acinetobacter johnsonii*	*Acinetobacter*	12F17	LC	Freshwater	-	-	-	KY611647
80	*Aeromonas veronii*	*Aeromonas*	12F18A	LC	Freshwater	weak	weak	weak	KY611648
81	*Citrobacter freundii*	*Citrobacter*	12F18B	LC	Freshwater	-	-	-	KY611649
82	*Brevundimonas nasdae*	*Brevundimonas*	12F19	LC	Freshwater	weak	-	-	KY611650
83	*Brevundimonas nasdae*	*Brevundimonas*	12F2	LC	Freshwater	-	-	-	KY611651
84	*Citrobacter freundii*	*Citrobacter*	12F20	LC	Freshwater	-	-	-	KY611652
85	*Brevundimonas nasdae*	*Brevundimonas*	12F6A	LC	Freshwater	-	-	-	KY611654
86	*Curtobacterium luteum*	*Curtobacterium*	12F7	LC	Freshwater	-	-	-	KY611656
87	*Microbacterium hydrothermale*	*Microbacterium*	12F9	LC	Freshwater	-	-	-	KY611658
88	*Comamonas koreensis*	*Comamonas*	17M1	BR1	Freshwater	-	-	-	KY611659
89	*Staphylococcus warneri*	*Staphylococcus*	17M10	BR1	Freshwater	-	-	-	KY611660
90	*Staphylococcus pasteuri*	*Staphylococcus*	17M3	BR1	Freshwater	-	-	-	KY611661
91	*Staphylococcus caprae*	*Staphylococcus*	17M5	BR1	Freshwater	-	-	-	KY611663
92	*Staphylococcus warneri*	*Staphylococcus*	17M6	BR1	Freshwater	-	-	-	KY611664
93	*Methylobacterium goesingense*	*Methylobacterium*	17M7	BR1	Freshwater	-	-	-	KY611665
94	*Aeromonas punctata subsp. punctata*	*Aeromonas*	17M8	BR1	Freshwater	moderate	moderate	moderate	-
95	*Bacillus drentensis*	*Bacillus*	20M10	BR1	Freshwater	-	-	-	KY611675
96	*Variovorax boronicumulans*	*Variovorax*	20M1a	BR1	Freshwater	-	-	-	KY611673
97	*Amphibiibacter pelophylacis*	*Amphibiibacter*	20M2A	BR1	Freshwater	-	-	-	KY611676
98	*Cellulosimicrobium funkei*	*Cellulosimicrobium*	20M3	BR1	Freshwater	-	-	weak	KY611678
99	*Phyllobacterium myrsinacearum*	*Phyllobacterium*	20M4	BR1	Freshwater	weak	weak	weak	KY611679
100	*Bacillus drentensis*	*Bacillus*	20M5A	BR1	Freshwater	-	-	-	KY611681
101	*Bacillus soli*	*Bacillus*	20M5B	BR1	Freshwater	-	-	-	KY611683
102	*Staphylococcus pasteuri*	*Staphylococcus*	20M6	BR1	Freshwater	-	-	-	KY611685
103	*Bacillus drentensis*	*Bacillus*	20M7	BR1	Freshwater	-	-	-	KY611686
104	*Phyllobacterium myrsinacearum*	*Phyllobacterium*	20M8	BR1	Freshwater	-	-	-	KY611687
105	*Stenotrophomonas rhizophila*	*Stenotrophomonas*	20M9	BR1	Freshwater	moderate	moderate	moderate	KY611688
106	*Curtobacterium flaccumfaciens*	*Curtobacterium*	22F1	BR2	Freshwater	-	-	-	KY611689
107	*Pantoea agglomerans*	*Pantoea*	22F10	BR2	Freshwater	-	-	-	KY611690

108	*Agrococcus jejuensis*	*Agrococcus*	22F13A	BR2	Freshwater	-	-	-	KY611692
109	*Acinetobacter baylyi*	*Acinetobacter*	22F13B	BR2	Freshwater	-	-	-	KY611693
110	*Acinetobacter soli*	*Acinetobacter*	22F13C	BR2	Freshwater	-	-	-	-
111	*Microbacterium trichothecenolyticum*	*Microbacterium*	22F14	BR2	Freshwater	-	-	-	KY611694
112	*Pantoea agglomerans*	*Pantoea*	22F15A	BR2	Freshwater	-	-	-	KY611695
113	*Rhizobium mesoamericanum*	*Rhizobium*	22F3	BR2	Freshwater	weak	-	-	KY611698
114	*Microbacterium foliorum*	*Microbacterium*	22F3a	BR2	Freshwater	moderate	moderate	moderate	KY611699
115	*Rhizobium mesoamericanum*	*Rhizobium*	22F6A	BR2	Freshwater	-	-	-	KY611701
116	*Rhizobium etli*	*Rhizobium*	22F6B1	BR2	Freshwater	-	-	-	KY611702
117	*Microbacterium foliorum*	*Microbacterium*	22F6B2	BR2	Freshwater	-	-	-	KY611703
118	*Lactococcus taiwanensis*	*Lactococcus*	22F6C	BR2	Freshwater	-	-	-	KY611704
119	*Lactococcus garvieae*	*Lactococcus*	22F8	BR2	Freshwater	-	-	-	KY611705
120	*Microbacterium oxydans*	*Microbacterium*	22F9	BR2	Freshwater	-	-	-	KY611706
121	*Staphylococcus pasteuri*	*Staphylococcus*	23M1	BR2	Freshwater	-	-	-	KY611707
122	*Acinetobacter junii*	*Acinetobacter*	23M19	BR2	Freshwater	-	-	-	KY611709
123	*Bacillus anthracis*	*Bacillus*	23M2A1	BR2	Freshwater	moderate	moderate	moderate	KY611710
124	*Acidovorax radicis*	*Acidovorax*	23M2B	BR2	Freshwater	moderate	moderate	moderate	KY611712
125	*Acinetobacter soli*	*Acinetobacter*	23M4	BR2	Freshwater	-	-	-	KY611714
126	*Pseudomonas azotoformans*	*Pseudomonas*	23M6	BR2	Freshwater	-	moderate	moderate	KY611715
127	*Yersinia enterocolitica subsp. palearctica*	*Yersinia*	23M7	BR2	Freshwater	-	-	-	KY611716
128	*Acidovorax radicis*	*Acidovorax*	23M8	BR2	Freshwater	-	-	-	KY611717
129	*Mycobacterium frederiksbergense DSM 44346(T)*	*Mycobacterium*	TP1_2	TP	Metal/Acid	-	-	-	KT720444
130	*Pseudomonas koreensis Ps 9-14(T)*	*Pseudomonas*	TP1_3	TP	Metal/Acid	moderate	moderate	moderate	KT720408
131	*Microbacterium testaceum DSM 2016*	*Microbacterium*	TP1_1	TP	Metal/Acid	-	-	-	KT720440
132	*Bacillus aerophilus 28K(T*	*Bacillus*	TP1_4	TP	Metal/Acid	moderate	moderate	moderate	KT720445
133	*Bacillus safensis FO-036b(T)*	*Bacillus*	TP1_5	TP	Metal/Acid	strong	moderate	moderate	KT720446
134	*Bacillus simplex NBRC 15720(T)*	*Bacillus*	TP1_6	TP	Metal/Acid	weak	weak	weak	KT720447
135	*Moraxella osloensis NCTC 10465(T)*	*Moraxella*	TP2_1	TP	Metal/Acid	-	-	-	KT720409
136	*Brevundimonas nasdae GTC 1043(T)*	*Brevundimonas*	TP2_2	TP	Metal/Acid	weak	weak	weak	KT720410
137	*Pseudomonas syringae ATCC 19310(T)*	*Pseudomonas*	TP2_4	TP	Metal/Acid	-	-	-	KT720412
138	*Moraxella osloensis NCTC 10465(T)*	*Moraxella*	TP2_5	TP	Metal/Acid	-	-	-	KT720413
139	*Paenibacillus typhae xj7(T) (JN256679)*	*Paenibacillus*	TP10_2	TP	Metal/Acid	-	-	-	KT720436
140	*Microbacterium testaceum DSM 20166(T)*	*Microbacterium*	TP10_5	TP	Metal/Acid	weak	weak	weak	KT720437
141	*Cellulomonas composti TR7-06(T)*	*Cellulomonas*	TP10_6	TP	Metal/Acid	-	-	-	KT720438
142	*Ensifer adhaerens LMG 20216(T)*	*Ensifer*	TP10_8	TP	Metal/Acid	weak	-	-	KT720406
143	*Cellulomonas fimi ATCC 484(T)*	*Cellulomonas*	TP10_10	TP	Metal/Acid	-	-	-	KT720435
144	*Hydrotalea flava CCUG 51397(T)*	*Hydrotalea*	TP3_2	TP	Metal/Acid	-	-	-	KT720414
145	*Micrococcus luteus NCTC 2665(T)*	*Micrococcus*	TP3_3	TP	Metal/Acid	-	-	-	KT720448

146	*Rhizobium rosettiformans W3(T)*	*Rhizobium*	TP3_1	TP	Metal/Acid	weak	moderate	weak	-
147	*Janibacter terrae CS12(T)*	*Janibacter*	TP3_5	TP	Metal/Acid	-	-	-	KT720449
148	*Leifsonia shinshuensis JCM 10591(T)*	*Leifsonia*	TP11_6	TP	Metal/Acid	weak	weak	weak	KT720443
149	*Sphingomonas cynarae SPC-1(T)*	*Sphingomonas*	TP11_2	TP	Metal/Acid	-	-	-	KT720407
150	*Bacillus indicus Sd/3(T*	*Bacillus*	TP11_1	TP	Metal/Acid	weak	-	-	KT720441
151	*Staphylococcus saprophyticus subsp. bovis*	*Staphylococcus*	TP11_4	TP	Metal/Acid	moderate	moderate	moderate	KT720442
152	*Stenotrophomonas rhizophila*	*Stenotrophomonas*	1M1	AF	Metal/Acid	-	-	-	KY611666
153	*Microbacterium maritypicum*	*Microbacterium*	1M2	AF	Metal/Acid	-	-	-	KY611667
154	*Enterobacter ludwigii*	*Enterobacter*	1M3	AF	Metal/Acid	weak	weak	weak	KY611668
155	*Enterobacter ludwigii*	*Enterobacter*	1M4	AF	Metal/Acid	weak	weak	weak	KY611669
156	*Stenotrophomonas rhizophila*	*Stenotrophomonas*	1M5A	AF	Metal/Acid	-	-	-	KY611670
157	*Stenotrophomonas rhizophila*	*Stenotrophomonas*	1M6	AF	Metal/Acid	-	-	-	KY611672
158	*Cellulomonas pakistanensis*	*Cellulomonas*	2M2B	AF	Metal/Acid	-	-	-	KY611718
159	*Cellulomonas pakistanensis*	*Cellulomonas*	2M3A	AF	Metal/Acid	-	-	-	KY611719
160	*Cellulomonas pakistanensis*	*Cellulomonas*	2M6	AF	Metal/Acid	-	-	-	KY611722
161	*Stenotrophomonas rhizophila*	*Stenotrophomonas*	3F10	AF	Metal/Acid	-	-	weak	KY611726
162	*Paenibacillus odorifer*	*Paenibacillus*	3F4	AF	Metal/Acid	-	-	-	KY611727
163	*Stenotrophomonas rhizophila*	*Stenotrophomonas*	3F6	AF	Metal/Acid	weak	-	-	KY611728
164	*Lactococcus taiwanensis*	*Lactococcus*	3F7	AF	Metal/Acid	-	-	-	KY611729
165	*Bacillus drentensis*	*Bacillus*	3F8	AF	Metal/Acid	moderate	moderate	moderate	KY611730
166	*Bacillus aerophilus*	*Bacillus*	3F-A	AF	Metal/Acid	moderate	moderate	moderate	KY611723
167	*Bacillus altitudinis*	*Bacillus*	3F-B	AF	Metal/Acid	strong	moderate	moderate	KY611724
168	*Serratia fonticola*	*Serratia*	4M1	AF	Metal/Acid	-	-	-	KY611731
169	*Staphylococcus pasteuri*	*Staphylococcus*	4M2	AF	Metal/Acid	-	-	-	KY611732
170	*Staphylococcus warneri*	*Staphylococcus*	4M3A	AF	Metal/Acid	-	-	-	KY611733
171	*Microbacterium paraoxydans*	*Microbacterium*	4M3B	AF	Metal/Acid	-	-	-	KY611734
172	*Serratia fonticola*	*Serratia*	4M4	AF	Metal/Acid	-	-	-	KY611735
173	*Serratia fonticola*	*Serratia*	4M5	AF	Metal/Acid	weak	weak	-	KY611736
174	*Erwinia persicina*	*Serratia*	5F1	AF	Metal/Acid	weak	-	-	KY611737
175	*Erwinia persicina*	*Serratia*	5F2	AF	Metal/Acid	weak	weak	-	KY611738
176	*Brevundimonas vesicularis*	*Brevundimonas*	5F5	AF	Metal/Acid	weak	-	weak	KY611740
177	*Erwinia persicina*	*Erwinia*	5F8	AF	Metal/Acid	weak	-	-	KY611743
178	*Erwinia persicina*	*Erwinia*	5F9	AF	Metal/Acid	weak	-	-	KY611744
179	*Raoultella planticola*	*Raoultella*	6M1	AF	Metal/Acid	-	-	-	KY611745
180	*Acinetobacter soli*	*Acinetobacter*	6M2	AF	Metal/Acid	-	-	-	KY611746
181	*Lactococcus fujiensis*	*Lactococcus*	6M3	AF	Metal/Acid	-	-	-	KY611747
182	*Acinetobacter soli*	*Acinetobacter*	6M4	AF	Metal/Acid	-	-	-	KY611748

183	*Acinetobacter soli*	*Acinetobacter*	6M5	AF	Metal/Acid	-	-	-	KY611749
184	*Acinetobacter soli*	*Acinetobacter*	6M6	AF	Metal/Acid	-	-	-	KY611750
185	*Serratia nematodiphila*	*Serratia*	7M1	AF	Metal/Acid	moderate	moderate	moderate	KY611751
186	*Bordetella petrii*	*Bordetella*	7M10	AF	Metal/Acid	-	-	-	KY611752
187	*Staphylococcus pasteuri*	*Staphylococcus*	7M2	AF	Metal/Acid	-	-	-	KY611753
188	*Herbaspirillum huttiense subsp. huttiense*	*Herbaspirillum*	7M4	AF	Metal/Acid	-	-	-	KY611754
189	*Staphylococcus pasteuri*	*Staphylococcus*	7M5	AF	Metal/Acid	-	-	-	KY611755
190	*Staphylococcus warneri*	*Staphylococcus*	7M6	AF	Metal/Acid	-	-	-	KY611756
191	*Cupriavidus metallidurans*	*Cupriavidus*	7M8	AF	Metal/Acid	-	-	-	KY611757
192	*Citrobacter youngae*	*Citrobacter*	8F1	AF	Metal/Acid	-	-	-	KY611759
193	*Citrobacter youngae*	*Citrobacter*	8F2	AF	Metal/Acid	-	-	-	KY611760
194	*Acinetobacter bohemicus*	*Acinetobacter*	8F3	AF	Metal/Acid	-	-	-	KY611761
195	*Raoultella planticola*	*Raoultella*	8F4	AF	Metal/Acid	-	-	-	KY611762
196	*Citrobacter freundii*	*Citrobacter*	8F5	AF	Metal/Acid	-	-	-	KY611763
