# Supplementary material for: Potential Protective Role of Amphibian Skin Bacteria Against Water Mold Saprolegnia spp
Source: J Fungi (Basel). 2025 Sep 2;11(9):649. doi: 10.3390/jof11090649 (PMC12471046; doi:10.3390/jof11090649)
Supplement: Supplementary file 1 [file jof-11-00649-s001.zip › S2-diferent media comparision.pdf]

Table S2- Bacterial isolates with antagonistic effect against *Saprolegnia* spp. comparing activity between R2A and PG.

#	closest EzTaxon (nº)	CODE	R2A			*PG*		
			***S. australis* AV**	***S. australis* SAP**	***S. diclina* SAP**	***S. australis* AV**	***S. australis* SAP**	***S. diclina* SAP**
1	*Bacillus arybhattai* B8W22	SL12_5	strong	strong	strong	moderate	moderate	moderate
2	*Bacillus aerophilus* 28K	SL12_7	moderate	moderate	strong	-	-	-
3	*Massilia aurea* AP13	SL2_9	moderate	moderate	moderate	weak	weak	weak
4	*Brevundimonas bullata* IAM 13153	SL3_9	moderate	-	-	weak	-	-
5	*Sphingomonas glacialis* C16y	SL3_8	moderate	-	-	weak	weak	weak
6	*Azorhizobium doebereinerae*	LB13_5	strong	strong	moderate	moderate	moderate	moderate
7	*Lapillicoccus jejuensis*	LB7_6	moderate	-	-	-	-	-
8	*Amphibiibacter pelophylacis*	LB1_7	moderate	-	-	-	-	-
9	*Acinetobacter guillouiae*	10M6A	moderate	moderate	moderate	moderate	moderate	moderate
10	*Serratia fonticola*	11M5	moderate	moderate	moderate	moderate	moderate	weak
11	*Aeromonas punctata subsp. punctata*	11M8B	moderate	moderate	weak	-	-	-
12	*Arthrobacter niigatensis*	12F1	moderate	weak	-	-	-	-
13	*Aeromonas punctata subsp. punctata*	17M8	moderate	moderate	moderate	moderate	moderate	weak
14	*Stenotrophomonas rhizophila*	20M9	moderate	moderate	moderate	moderate	moderate	weak
15	*Microbacterium foliorum*	22F3a	moderate	moderate	moderate	-	-	-
16	*Bacillus anthracis*	23M2A1	moderate	moderate	moderate	-	-	weak
17	*Acidovorax radicis*	23M2B	moderate	moderate	moderate	moderate	moderate	moderate
18	*Pseudomonas meridiana*	9F11	strong	strong	strong	weak	moderate	moderate
19	*Pseudomonas koreensis Ps 9-14(T)*	TP1_3	moderate	moderate	moderate	-	-	-
20	*Bacillus aerophilus 28K(T)*	TP1_4	moderate	moderate	moderate	-	-	-
21	*Bacillus safensis FO-036b(T)*	TP1_5	strong	moderate	moderate	-	-	-
22	*Staphylococcus saprophyticus subsp. bovis GTC 843(T)*	TP11_4	moderate	moderate	moderate	-	-	-
23	*Bacillus drentensis*	3F8	moderate	moderate	moderate	-	-	-
24	*Bacillus aerophilus*	3F-A	moderate	moderate	moderate	weak	-	-
25	*Bacillus altitudinis*	3F-B	strong	moderate	moderate	-	-	-
26	*Serratia nematodiphila*	7M1	moderate	moderate	moderate	strong	strong	moderate
